# Supplementary material for: Mitochondrial calcium exchange links metabolism with the epigenome to control cellular differentiation
Source: Nat Commun. 2019 Oct 4;10:4509. doi: 10.1038/s41467-019-12103-x (PMC6778142; doi:10.1038/s41467-019-12103-x)

Supplemental Figures:

Mitochondrial Calcium Exchange links Metabolism with the Epigenome to Control Cellular Differentiation

Alyssa A. Lombardi et al. *Nature Communications*. 2019

Supplemental Figure 1 – related to Figure 1.

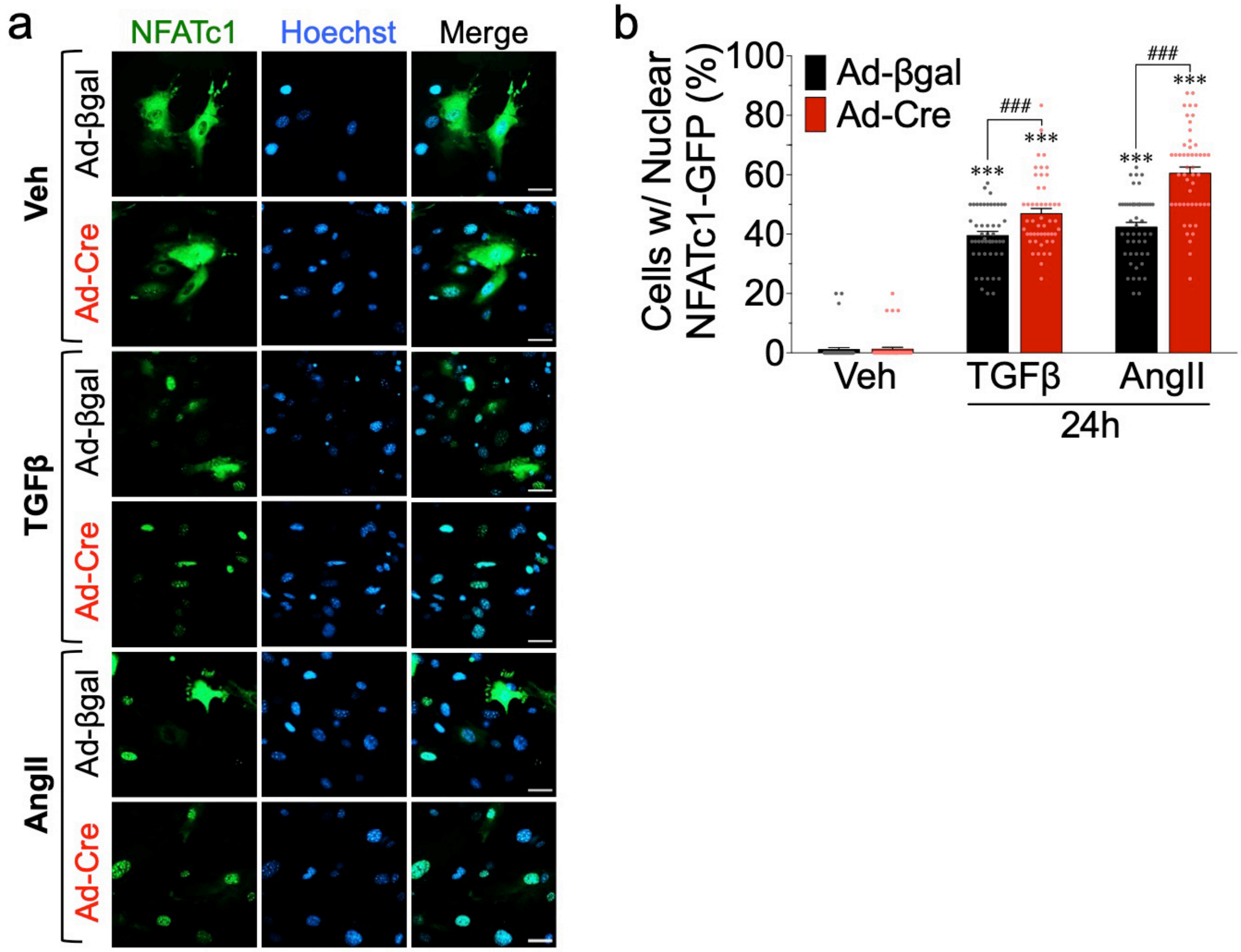

Supplemental Figure 1 – related to Figure 1. Loss of $_mCa^{2+}$ uptake enhances cytosolic signaling. a,b) *Mcu*$^{-/-}$ (Ad-Cre) and control (Ad-βgal) MEFs were transduced with adenovirus-encoding NFATc1-GFP and 24h later treated with TGFβ or AngII for 24h. Cells were stained with Hoechst to demarcate nuclei and imaged on a fluorescence microscope. Representative images are shown. Percentage of cells with nuclear NFATc1 was quantified. n=3 per group each with 50 cells quantified. *All data shown as mean ± SEM. ***p<0.001 vs. vehicle control analyzed by ANOVA. ###p<0.001 vs. Ad-βgal analyzed by t-test. Scale bar = 50µm.*

Supplemental Figure 2 – related to Figure 2.

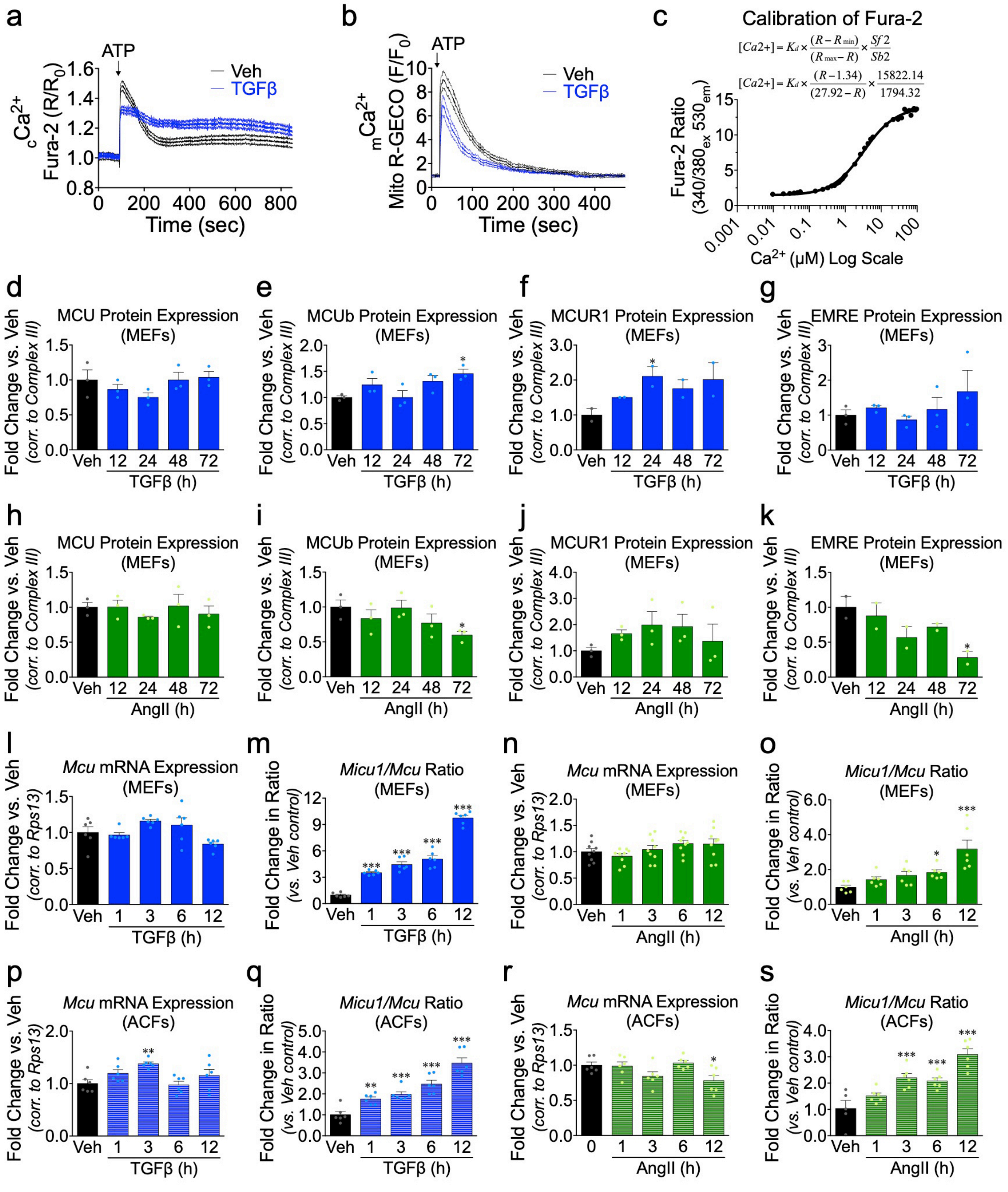

Supplemental Figure 2 – related to Figure 2. Calibration of Fura-2 Ca^{2+} reporter and quantification of expression of mtCU components post-TGFβ or AngII. a) WT MEFs plus or minus (+/-) 12h TGFβ were loaded with the Ca^{2+}-sensitive dye Fluo-4 AM. Fluorescence was recorded while 1mM ATP was delivered to initiate purinergic receptor-mediated IP3R Ca^{2+} release. n=28 cells. **b)** WT MEFs +/- 12h TGFβ were transduced with adenovirus encoding the mitochondrial calcium sensor, Mito R-GECO and fluorescence was recorded during 1mM ATP treatment. n=24 cells. **c)** Fura-2 was calibrated by the generation of a standard curve of Ca^{2+} (0.01-100μm) in experimental intracellular buffer to quantify actual Ca^{2+} content as shown in Figure 2c. Fura-2 fluorescence ratio was converted to [Ca^{2+}] by the following equation: [Ca^{2+}] = K_d * (R-R_{min})/(R_{max}-R) * Sf2/Sb2. (R_{min}= ratio in 0- Ca^{2+}; R_{max} = ratio at saturation; Sf2=380/510 reading in 0-Ca^{2+}; Sb2=380/510 reading with Ca^{2+} saturation. **d-k)** Fold change in expression of mtCU components from immunoblots in Figure 2h,m. Band density analysis was corrected to the loading control, Complex III (subunit UQCRC2). n=3 samples. **l-o)** Fold change in mRNA expression of *Micu1* and the ratio of *Micu1/Mcu* in MEFs. *Rps13* was used as the housekeeping gene; n=6. **p-s)** Fold change in mRNA expression of *Micu1* and the ratio of *Micu1/Mcu* in mouse adult cardiac fibroblasts (ACFs). *Rps13* was used as the housekeeping gene; n=6 samples. *Ca^{2+} traces: solid line = mean, dashed line = SEM. All data shown as mean ± SEM. ***p<0.001, **p<0.01, *p<0.05 vs. vehicle control analyzed by ANOVA.*

Supplemental Figure 3 – related to Figure 3.

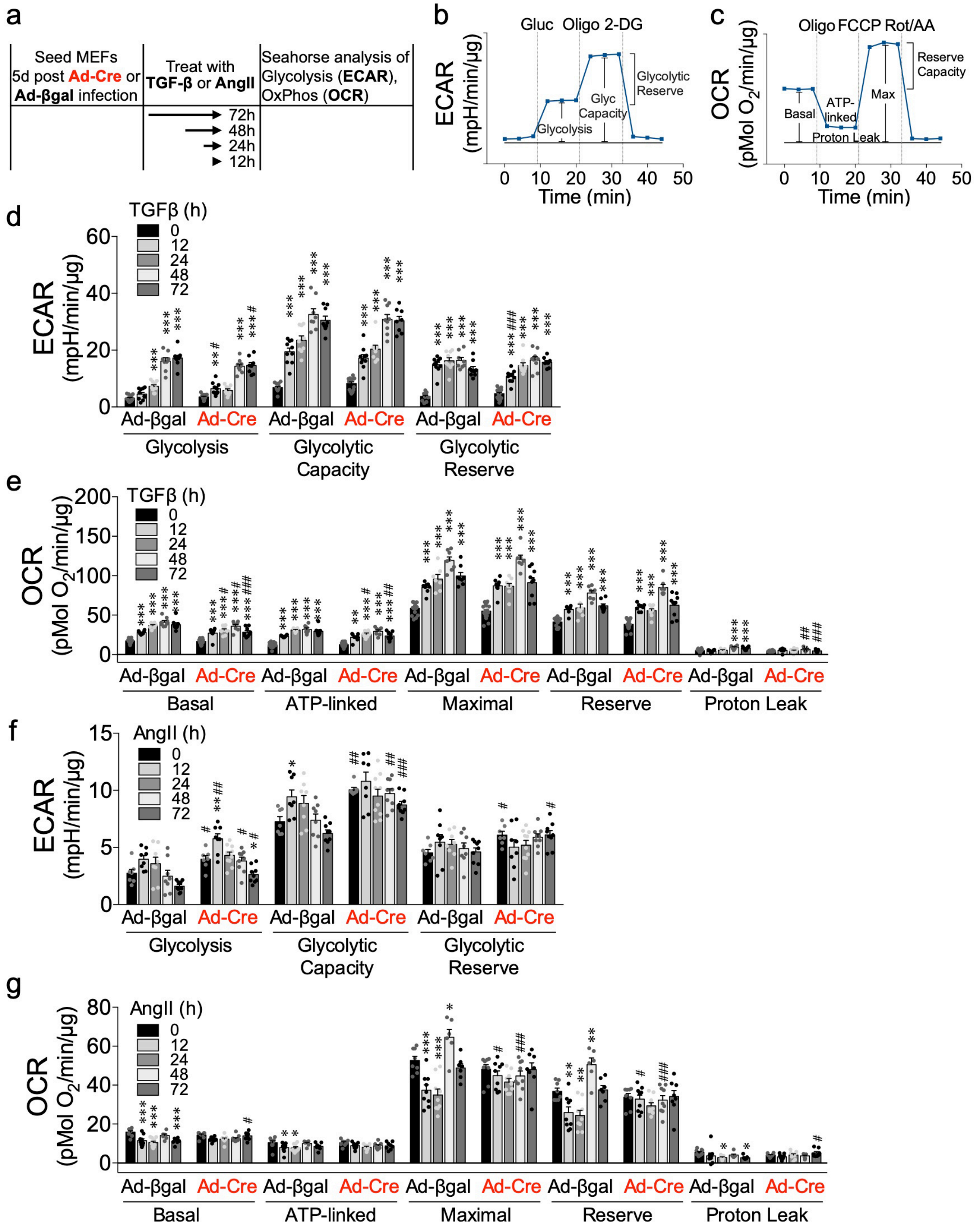

Supplemental Figure 3 – related to Figure 3. Seahorse analysis of glycolysis and oxidative phosphorylation. a-c) Schematic of experimental timeline. MEFs were treated with fibrotic stimuli for 12, 24, 48, or 72h and assayed for Glycolysis and Oxidative Phosphorylation using a Seahorse XF96 analyzer to measure extracellular acidification rates (ECAR, glycolysis) or oxygen consumption rates (OCR, OxPhos). **d)** Quantification of glycolysis, glycolytic capacity, and glycolytic reserve in *Mcu*$^{-/-}$ (Ad-Cre) and control (Ad-βgal) MEFs post-TGFβ. For Ad-βgal, n = 14 (t0), 10 (t12), 9 (t24), 8 (t48) and 9 (t72). For Ad-Cre, n = 15 (t0), 10 (t12), 9 (t24), 9 (t48) and 8 (t72). **e)** Quantification of basal respiration, ATP-linked respiration, maximal respiration, reserve capacity, and proton leak in *Mcu*$^{-/-}$ and control MEFs post-TGFβ. For Ad-βgal, n = 18 (t0), 8 (t12), 7 (t24), 8 (t48) and 8 (t72). For Ad-Cre, n = 18 (t0), 8 (t12), 9 (t24), 7 (t48) and 9 (t72). **f)** Quantification of glycolysis, glycolytic capacity, and glycolytic reserve in *Mcu*$^{-/-}$ and control MEFs post-AngII. For Ad-βgal, n = 7 (t0), 8 (t12), 8 (t24), 8 (t48) and 9 (t72). For Ad-Cre, n = 7 (t0), 8 (t12), 10 (t24), 9 (t48) and 9 (t72). **g)** Quantification of basal respiration, ATP-linked respiration, maximal respiration, reserve capacity, and proton leak in *Mcu*$^{-/-}$ and control MEFs post-AngII. For Ad-βgal, n = 8 (t0), 9 (t12), 9 (t24), 6 (t48) and 7 (t72). For Ad-Cre, n = 8 (t0), 9 (t12), 9 (t24), 9 (t48) and 8 (t72).. *All data shown as mean ± SEM. *** p<0.001, ** p<0.01, * p<0.05 vs. vehicle control analyzed by ANOVA. ### p<0.001, ## p<0.01, # p<0.05 vs. Ad-βgal analyzed by t-test.*

Supplemental Figure 4 – related to Figure 3.

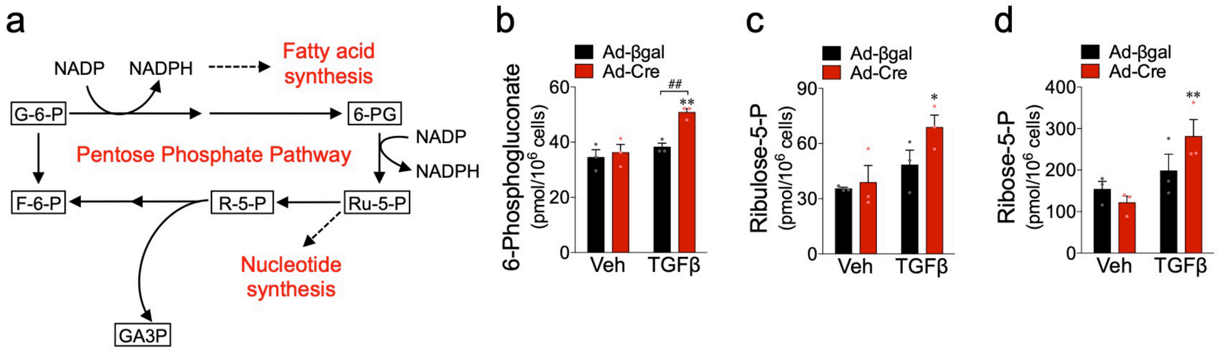

Supplemental Figure 4 – related to Figure 3. Quantification of metabolites involved in the pentose phosphate pathway. a) Schematic of the pentose phosphate pathway: glucose-6-phosphate (G-6-P), 6-phosphogluconate (6-PG), ribulose-5-phosphate (Ru-5-P), ribose-5-phosphate (R-5-P), glyceraldehyde-3-phosphate (GA3P), fructose-6-phosphate (F-6-P). **b-d)** Absolute concentration of pentose phosphate pathway metabolites 6-phosphogluconate, ribulose-5-P phosphate, and ribose-5-phosphate; n=3/group. *All data shown as mean ± SEM. ***p<0.001, **p<0.01, *p<0.05 vs. vehicle control analyzed by ANOVA. ###p<0.001, ##p<0.01, #p<0.05 vs. Ad-βgal analyzed by t-test.*

Supplemental Figure 5 – related to Figures 3 and 4.

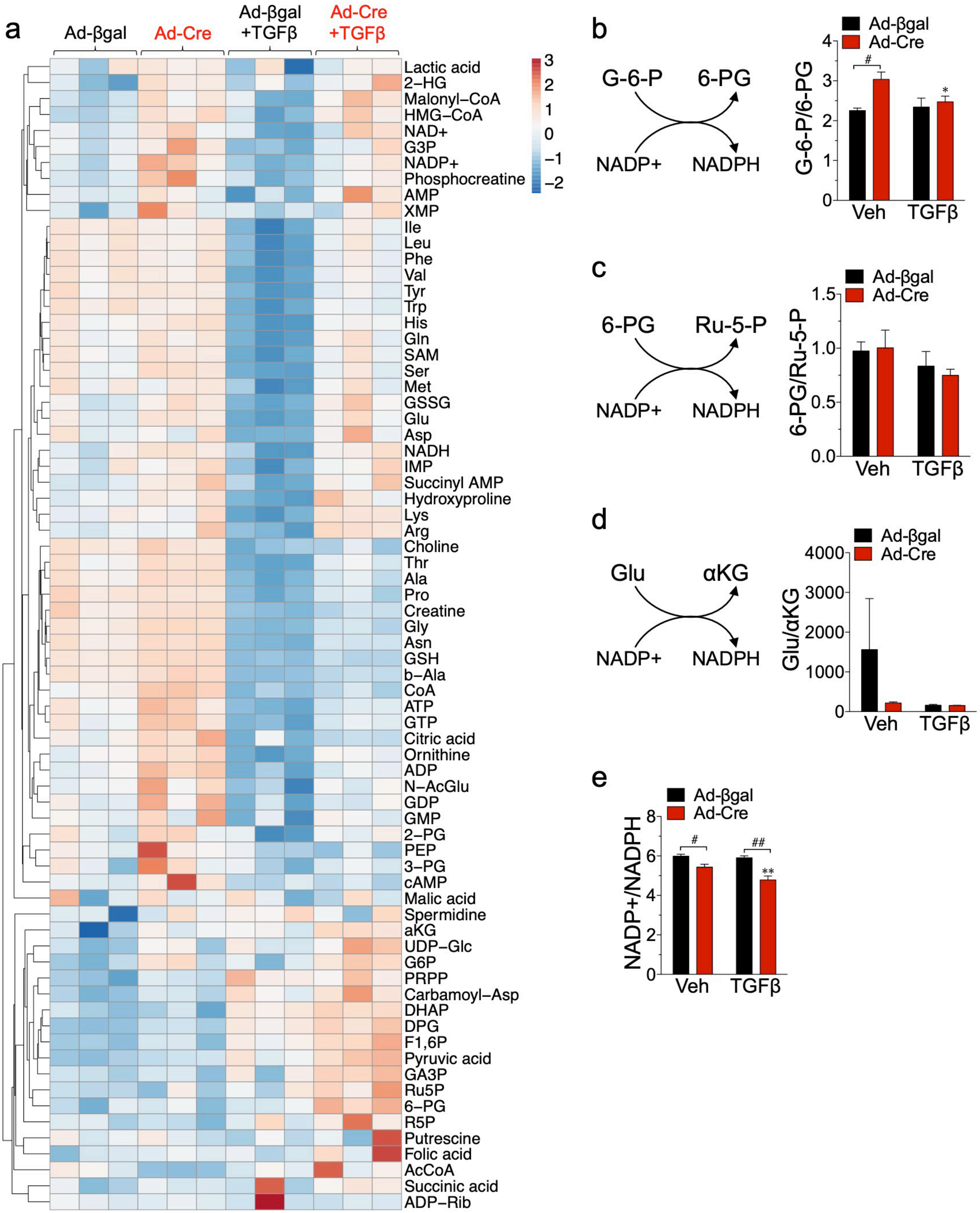

Supplemental Figure 5 – **related to Figures 3 and 4. a)** Heat map of metabolites. Heat map representation of metabolome profiles of *Mcu*$^{-/-}$ (Ad-Cre) and control (Ad-βgal) MEFs at baseline and post-TGFβ (12h). Unit variance scaling is applied to rows. Rows are clustered using Manhattan distance and average linkage. *Also see Supplemental Data File 1.* **b-e)** Ratios of metabolites involved in enzymatic reactions that require NADP+ as a cofactor, derived from absolute metabolite concentrations. An observed change in the metabolite ratio provides potential insight into the redox state of the cell, which could affect the interconversion of these metabolites; n=3. **b)** glucose-6-phosphate (G-6-P)/6-phosphogluconate (6-PG), **c)** 6-PG/ribulose-5-phosphate (Ru-5-P), **d)** Glutamate (Glu)/α-ketoglutarate (αKG), **e)** Cellular redox state (NADP+/NADPH ratio) examined by luciferin-based assay in WT and *Mcu*$^{-/-}$ MEFs ± 24h TGFβ; n=5. These data are suggestive that mtCU-Ca^{2+} uptake has a greater impact on mitochondrial NADPH redox than cytosolic redox state. *All data shown as mean ± SEM. **p<0.01, *p<0.05 vs. vehicle control analyzed by ANOVA. ##p<0.01, #p<0.05 vs. Ad-βgal analyzed by t-test.*

Supplemental Data File 1 – related to Figures 3 and 4. Absolute concentration of metabolites; n=3. *All data shown as mean ± SEM. (see excel file)*

Supplemental Figure 6 – related to Figure 5.

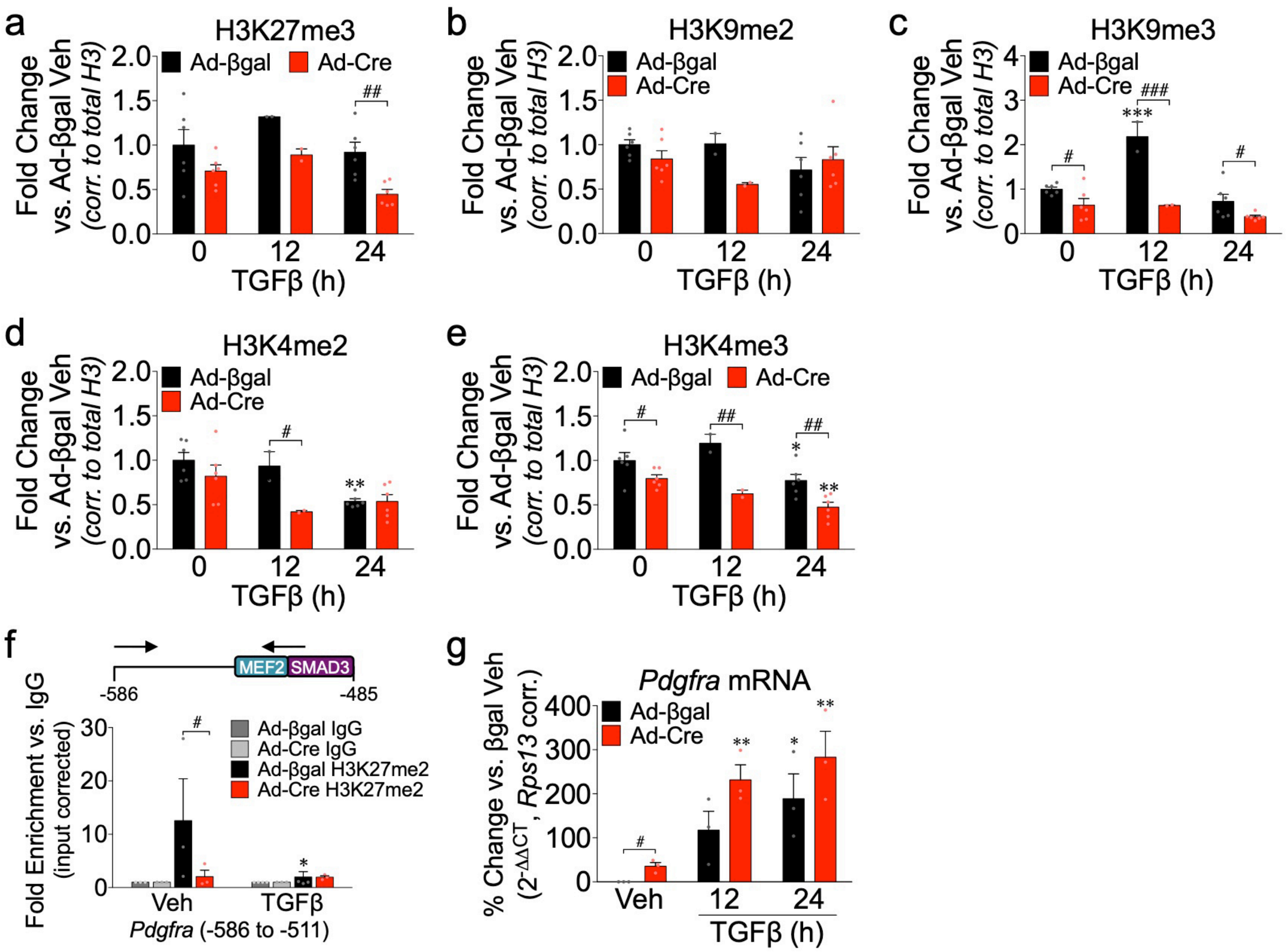

Supplemental Figure 6 – related to Figure 5. Quantification of H3K methylation.
a-e) MEFs were treated with TGFβ for 0, 12 or 24h and cell lysates were immunoblotted for specific methylated histone 3 lysine (H3K) residues – H3K27me3, H3K9me2, H3K9me3, H3K4me2, H3K4me3. Total H3 was used as a nuclear loading control and tubulin served as a total lysate loading control. Band density was normalized to total H3. **f)** H3K27me2 ChIP-qPCR of *platelet-derived growth factor receptor alpha (Pdgfra)* in *Mcu*$^{-/-}$ (Ad-Cre) and control (Ad-βgal) MEFs at baseline (veh) and following 12h TGFβ. Schematic shows loci of qPCR primers in relationship to myofibroblast transcription factor binding sites – MEF2 (myocyte enhancer factor 2), SMAD3 (SMAD family member 3). **g)** qPCR of *Pdgfra* mRNA in *Mcu*$^{-/-}$ (Ad-Cre) and control (Ad-βgal) MEFs at baseline (veh) and post-TGFβ. n=3 experiments for all quantified data. *All data shown as mean ± SEM. ***p<0.001, **p<0.01, *p<0.05 vs. vehicle control analyzed by ANOVA. ###p<0.001, ##p<0.01, #p<0.05 vs. Ad-βgal analyzed by t-test.*

Supplemental Figure 7 – related to Figure 5.

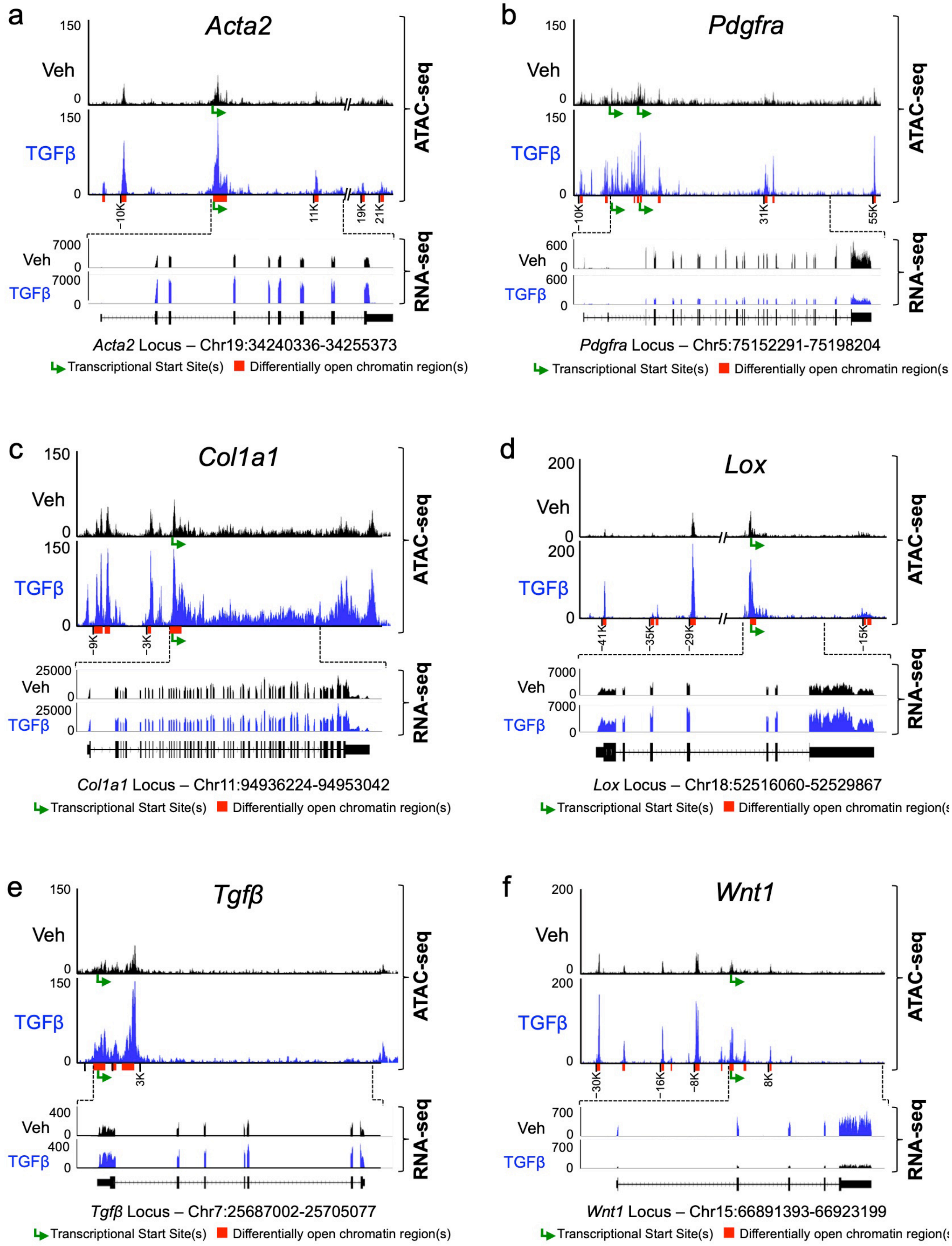

Supplemental Figure 7 – related to Figure 5. ATAC-seq and RNA-seq data. a-e) WT MEFs were treated with vehicle or TGFβ and assessed for chromatin accessibility and transcription using ATAC-seq and RNA-seq. Results of key myofibroblast gene loci are shown – α-smooth muscle actin (*Acta-2*), platelet-derived growth factor receptor alpha (*Pdgfra*), collagen type I alpha 1 chain (*Col1a1*), lysyl oxidase (*Lox*), transforming growth factor β (*Tgfβ*), and wnt family member 1 (*Wnt1*). The height of the genome browser tracks shows the number of reads normalized by read depth and overall peak enrichment in the library.

Supplemental Figure 8 – related to Figure 6.

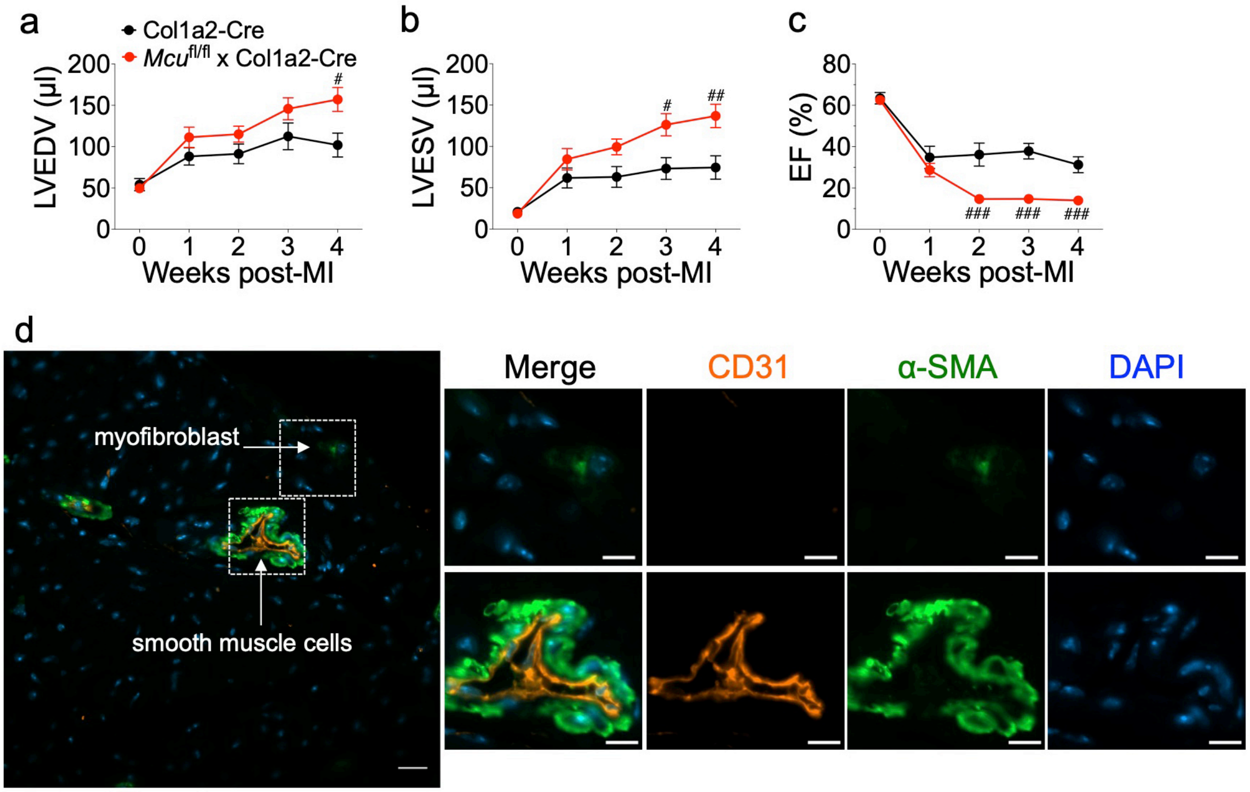

Supplemental Figure 8 – related to Figure 6. Echocardiographic parameters and representative immunohistochemistry images of myofibroblast identification. **a-c)** *Mcu*$^{fl/fl}$ x Col1a2-CreERT and control Col1a2-CreERT mice were treated with tamoxifen (40mg/kg/day) for 10d and allowed to rest 10d before permanent ligation of the left coronary artery. Cardiac function was analyzed by echocardiography 1wk prior to MI and every week thereafter. M-mode echo measurements of left ventricular end diastolic volume (LVEDV), left ventricular end systolic volume (LVESV), and ejection fraction (EF) were acquired. n=10 Col1a2-Cre, n=20 Mcu$^{fl/fl}$ x Col1a2-Cre. **d)** To quantify myofibroblasts, tissue sections were stained with anti-α-SMA, anti-CD31 (endothelial cell marker), and DAPI. Representative immunohistochemistry images show identification of myofibroblasts (α-SMA+/CD31-) vs. smooth muscle cells (α-SMA+/CD31+). *All data shown as mean ± SEM. ###p<0.001, ##p<0.01, #p<0.05 vs. Ad-βgal analyzed by t-test*. Large image *left*: *scale bar = 20μm, small inset images right = 10μm. Also see Supplemental Table 1.*

Supplemental Table 1 – related to Figure 6. Echocardiographic results of left-ventricular (LV) function at baseline (wk 0) and post-MI. All values were collected from M-mode analysis to measure intraventricular septum thickness (IVS) during diastole (IVS;d) and systole (IVS;s), left-ventricular end-diastolic diameter (LVEDD), left-ventricular end-systolic diameter (LVESD), left-ventricular end-diastolic posterior wall thickness (LVEDPW), left-ventricular end-systolic posterior wall thickness (LVESPW), ejection fraction (EF), fractional shortening (FS), left-ventricular end-diastolic volume (LVEDV), and left-ventricular end-systolic volume (LVEDV). *All data shown as mean ± SEM. ***p<0.001, **p<0.01, *p<0.05 vs. wk 0 analyzed by ANOVA. ###p<0.001, ##p<0.01, #p<0.05 vs. Ad-βgal analyzed by t-test.*

	Wks post-MI	n/group	IVS;d (mm)	IVS;s (mm)	LVEDD (mm)	LVESD (mm)	LVEDP (mm)	LVESP (mm)	EF (%)	FS (%)	LVEDV (µl)	LVESV (µl)
Col1a2-Cre	0	10	0.69±0.07	1.00±0.09	3.53±0.18	2.35±0.17	0.68±0.03	0.96±0.07	63.43±2.74	33.94±1.92	54.11±7.41	20.81±4.29
	1	10	0.70±0.04	0.90±0.06	4.35±0.22	3.66±0.30 **	0.71±0.05	0.92±0.10	34.83±5.36 ***	16.95±2.81 ***	88.12±10.45	61.86±12.00
	2	10	0.69±0.04	0.94±0.08	4.40±0.24	3.67±0.32 **	0.72±0.07	0.85±0.09	36.18±5.56 ***	17.79±3.03 ***	91.32±11.98	63.14±12.63
	3	10	0.67±0.06	0.91±0.09	4.80±0.29 **	3.94±0.30 ***	0.67±0.05	0.87±0.08	37.80±3.78 ***	18.47±2.01 ***	112.43±16.19 *	73.19±13.27
	4	10	0.68±0.04	1.00±0.08	4.60±0.27 *	3.96±0.32 ***	0.78±0.08	0.87±0.08	31.25±3.87 ***	14.86±1.98 ***	101.94±14.43	74.53±14.25 *
Mcu$^{fl/fl}$ x Col1a2-Cre	0	20	0.90±0.03 ##	1.27±0.04	3.44±0.07	2.31±0.06	0.87±0.06	1.18±0.07	62.54±1.24	33.06±0.88	49.52±2.41	18.71±1.20
	1	20	0.66±0.02 ***	0.93±0.06 ***	4.77±0.21 ***	4.16±0.25 ***	0.76±0.05	0.90±0.06 **	28.72±3.28 ***	13.71±1.65 ***	111.30±12.27 ***	84.58±12.95 ***
	2	20	0.54±0.03 ***	0.67±0.05 *** #	4.88±0.16 ***	4.56±0.17 ***	0.60±0.06 **	0.69±0.06 ***	14.68±1.75 *** ###	6.63±0.83 *** ###	115.12±9.72 ***	99.42±9.53 ***
	3	20	0.61±0.04 ***	0.78±0.07 ***	5.39±0.19 ***	5.04±0.21 *** ##	0.64±0.06 **	0.68±0.06 ***	14.71±2.03 *** ###	6.73±0.97 *** ###	145.79±13.30 ***	126.27±13.46 *** #
	4	20	0.62±0.05 ***	0.72±0.07 *** #	5.55±0.23 *** #	5.21±0.24 *** ##	0.61±0.05 **	0.67±0.04 ***	13.96±1.95 *** ###	6.39±0.94 *** ##	157.11±14.40 *** #	136.95±14.26 *** ##

Supplemental Figure 9. Western blots related to Figure 1.

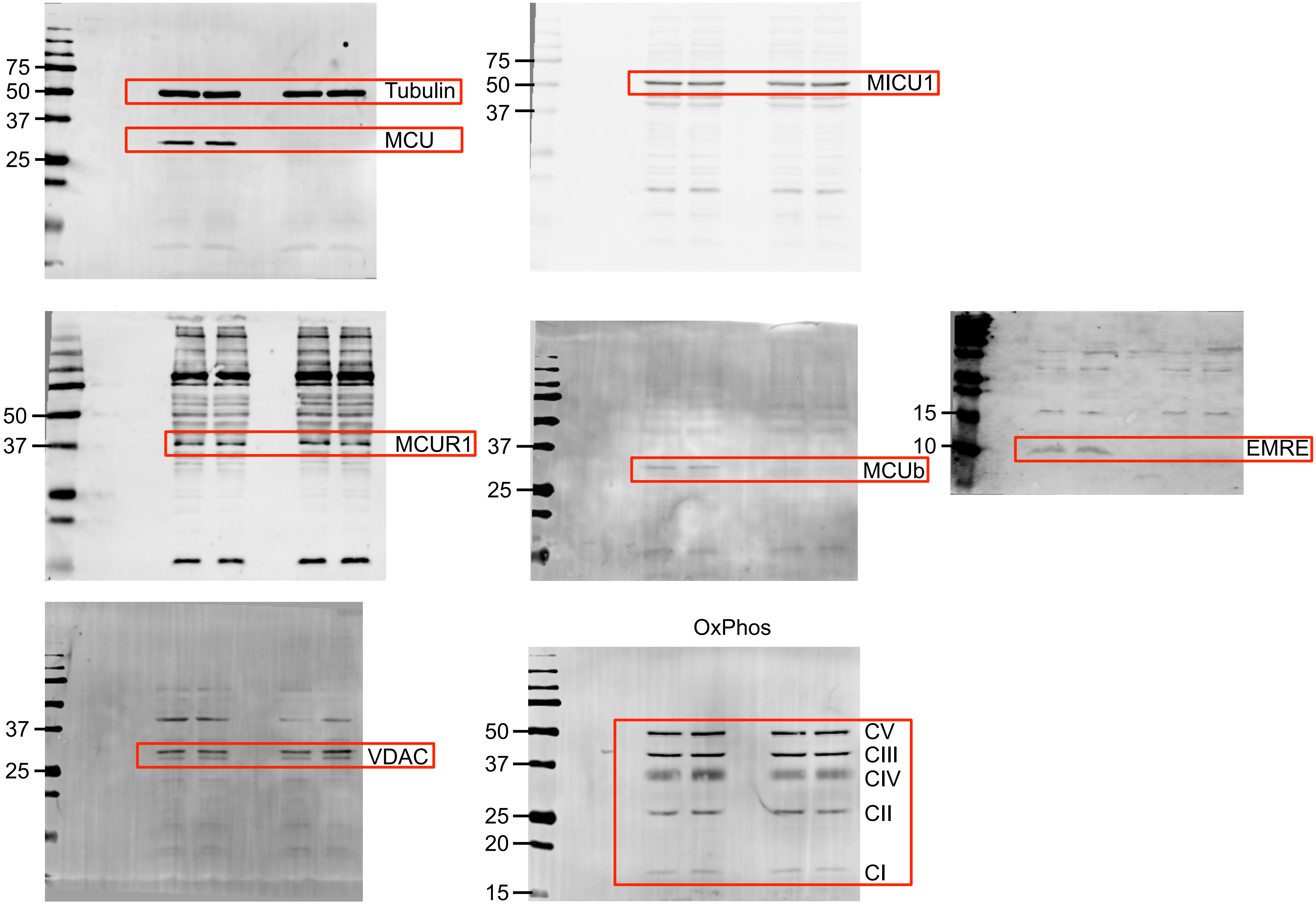

Supplemental Figure 10. Western blots related to Figure 2.

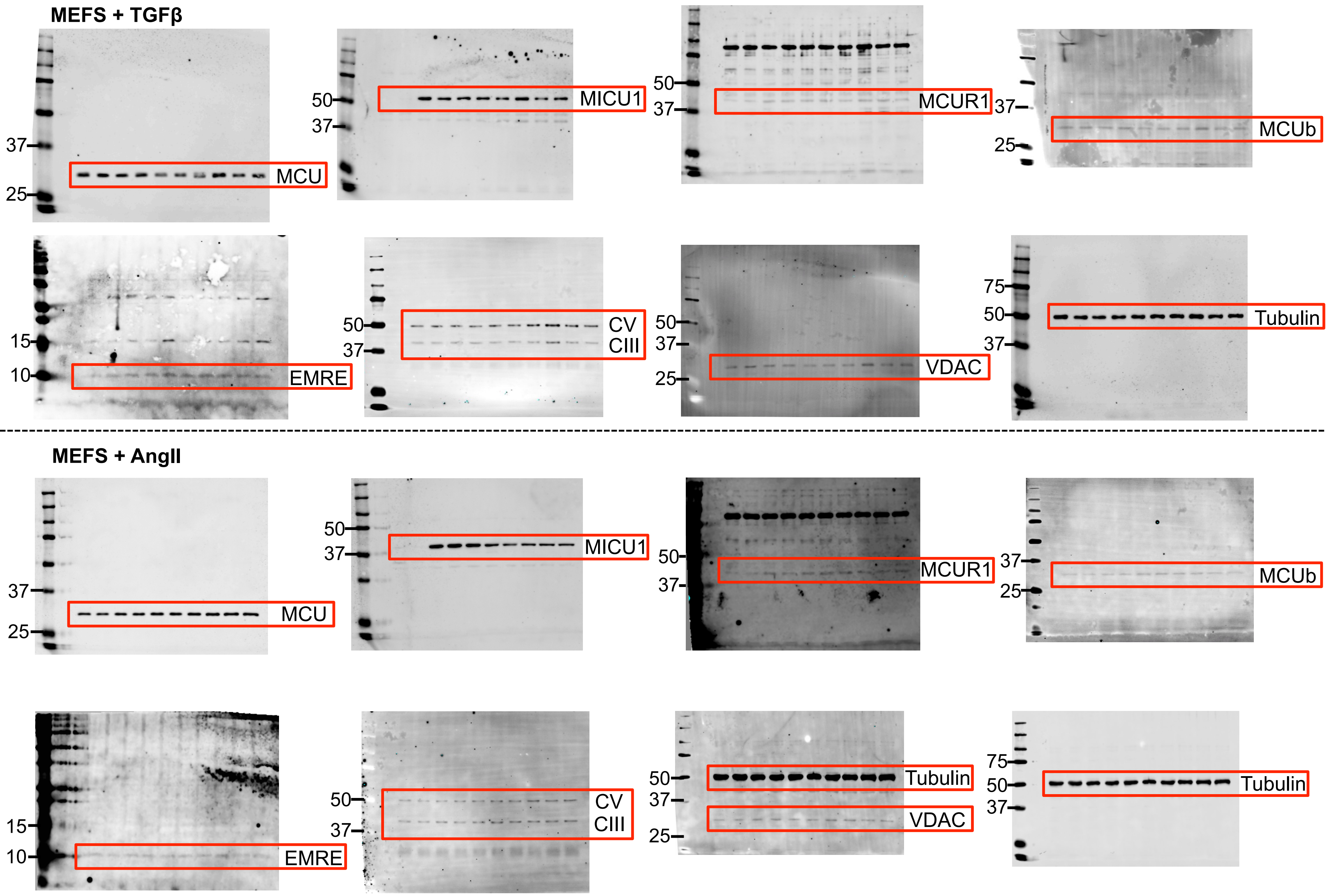

Western blots related to Figure 2 continued.

MEFS + TGFβ

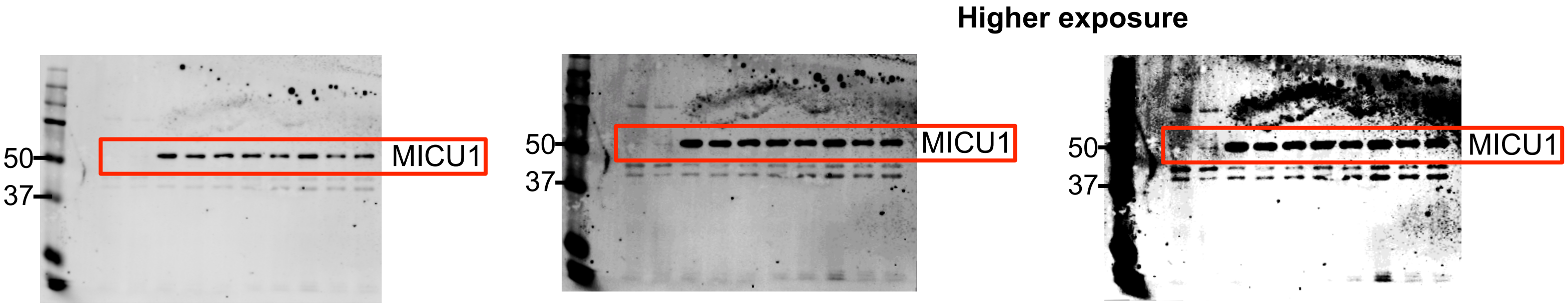

MEFS + AngII

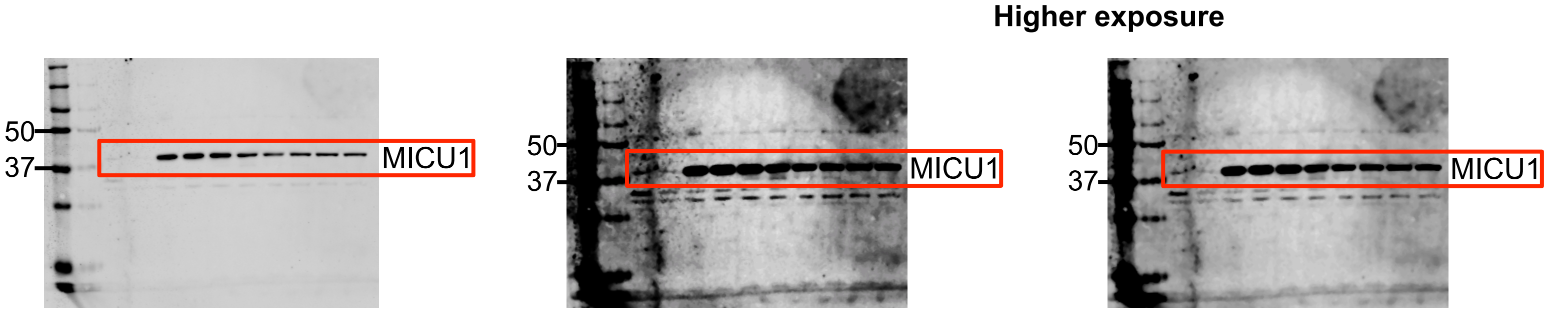

Supplemental Figure 11. Western blots related to Figure 4.

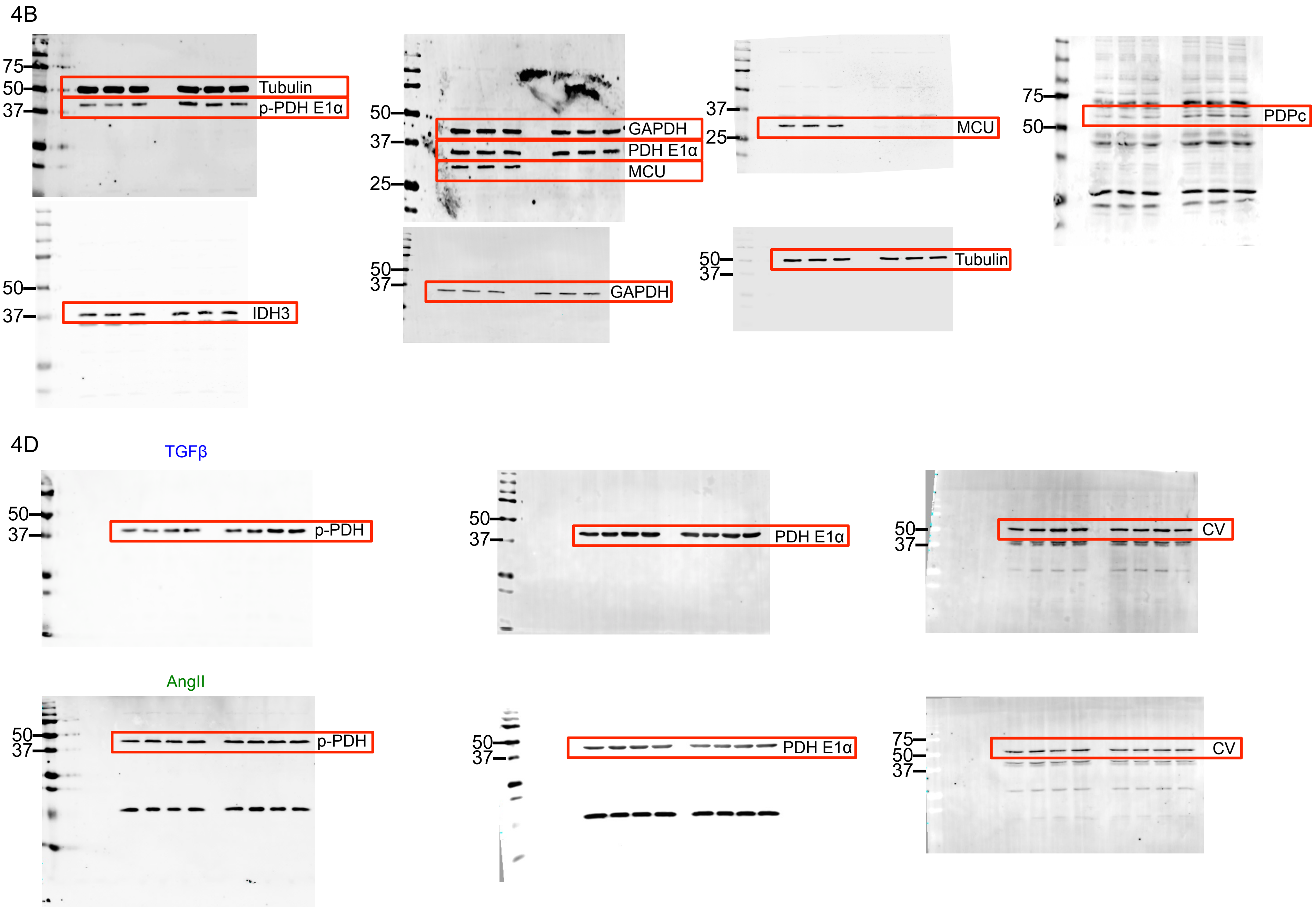

Supplemental Figure 12. Western blots related to Figure 5.

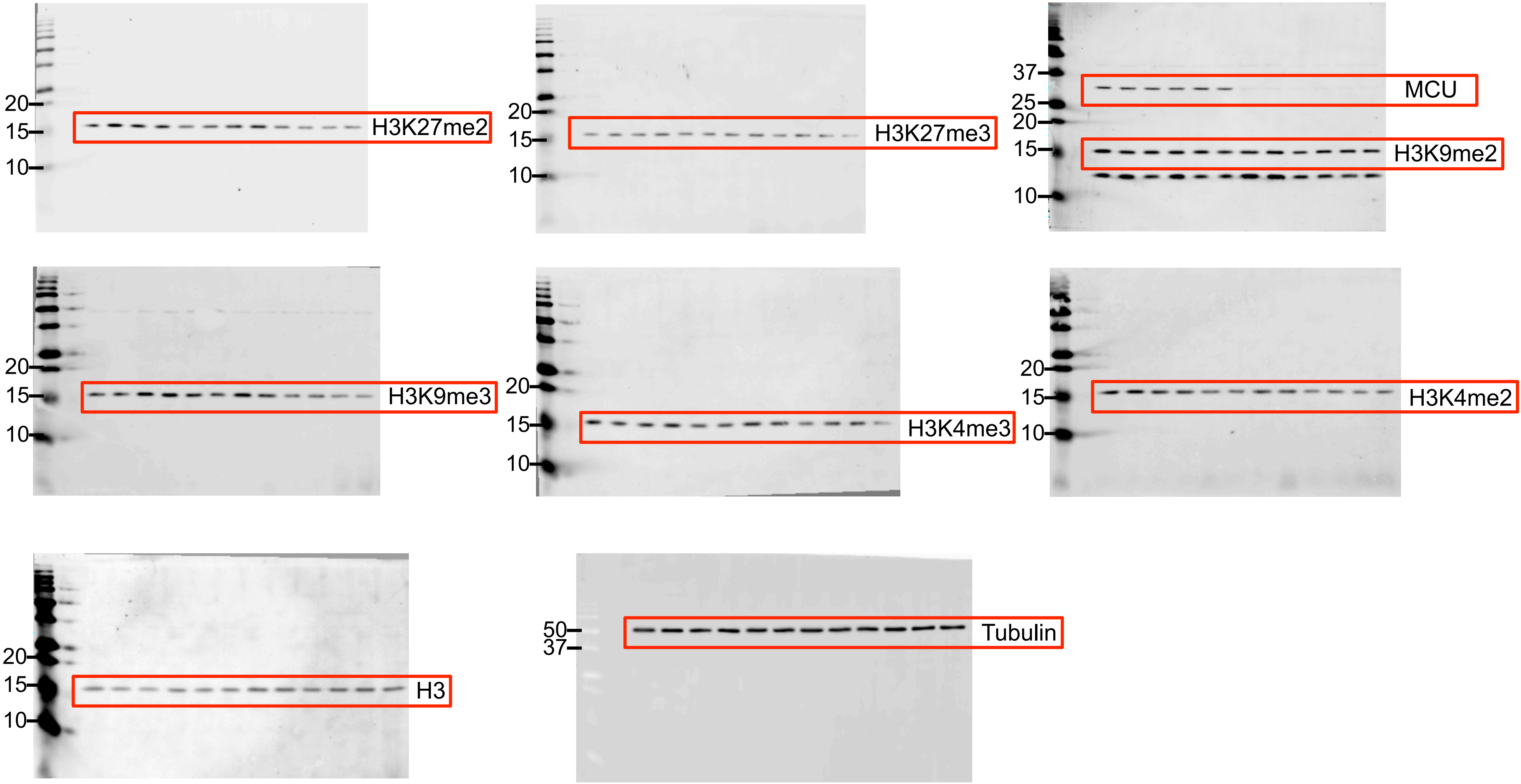

Supplemental Figure 13. Western blots related to Figure 6.

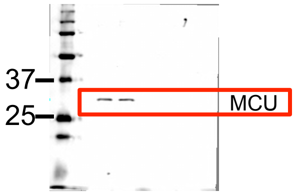

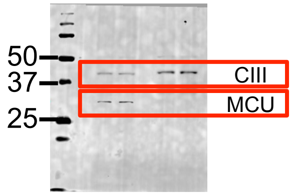

Supplement: Supplementary file 1 — Supplementary Information [file 41467_2019_12103_MOESM1_ESM.pdf]
